# Supplementary material for: Polymorphisms in Pfkelch13 domains before and after the introduction of artemisinin-based combination therapy in Southwest Nigeria
Source: PLoS One. 2025 Mar 31;20(3):e0316479. doi: 10.1371/journal.pone.0316479 (PMC11957316; doi:10.1371/journal.pone.0316479)

Samples: 13954
Bases: 1063
Average spacing: 14

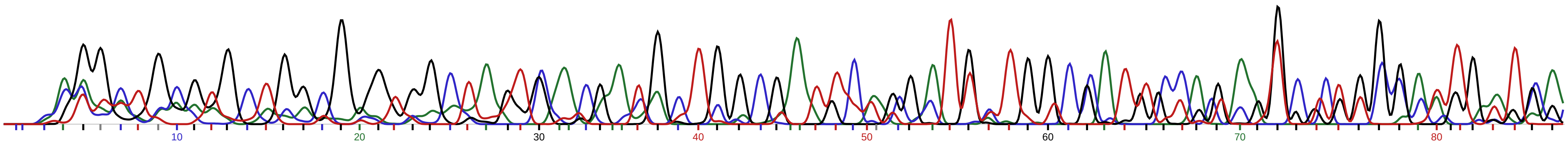

CC C A G - C T - C G T G C T G G C G A G T G G C T A GT G AA C GAA T G C T G G C GAA T T C T- CG A T G T T G G C G A T G G T G G A G G G T T G G G A T GTG T T G G

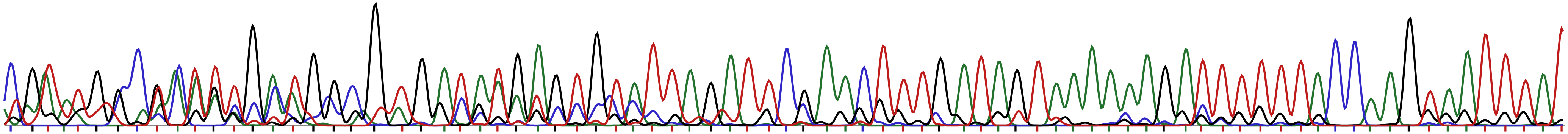

C G T A T G G C T A T G T G A T G G G G T G A T A T G A G T G T A T T A G A T T C G A A C T T T G A T A G T A A A A A A G A T T T T T T A C C A A G T A A T T T A T

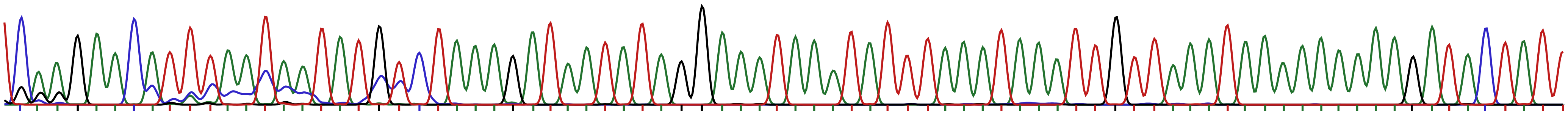

C A A G A A C A T T T A A T A A T A T G T C T A A A G A T A A T A T A G G A A A T A A A T A T T T A A A T A A A T T G T T A A A T A A A A A A A A A G A T A C T A T T

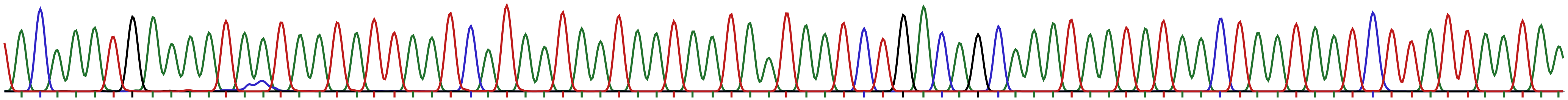

A C A A A T G A A A A T A A T A A T A T T A A T C A T A A T A A T A A T A A T A A T A A T C T G A C A G C A A A T A A T A T A A C T A A T A A T C T T A T T A A T A A

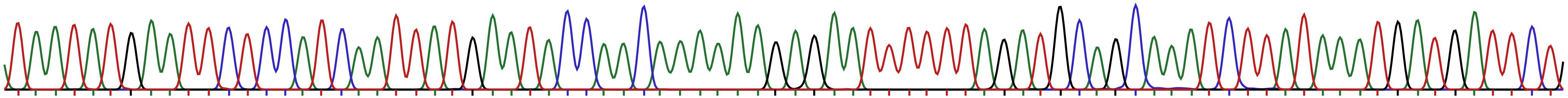

T A A T A T G A A T T C T C C A T C A A T T A T G A A T A C C A A C A A A A A A G A G A A T T T T T T A G A T G C A G C A A A T C T T A T A A A T G A T G A T T C T

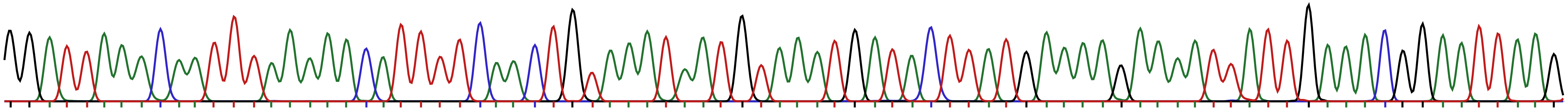

G G A T T A A A C A A T T T A A A A A C A T T T T C A A C T G T A A A T A A T G T A A A T G A T A C T T A T G A A A A G A A A A T T A T T G A A A C G G A A T T A A G

Samples: 13954
Bases: 1063
Average spacing: 14

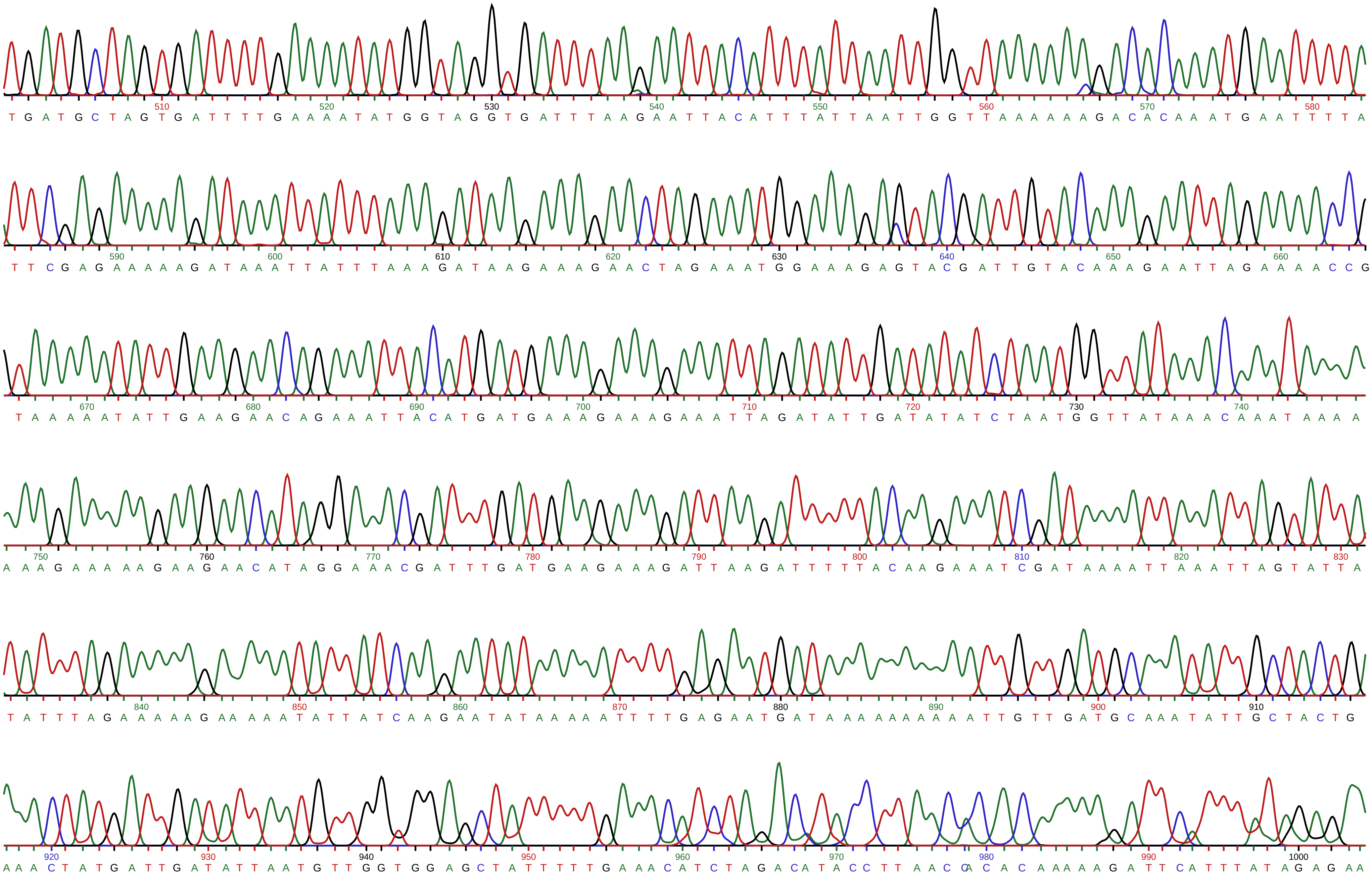

Samples: 13954
Bases: 1063
Average spacing: 14

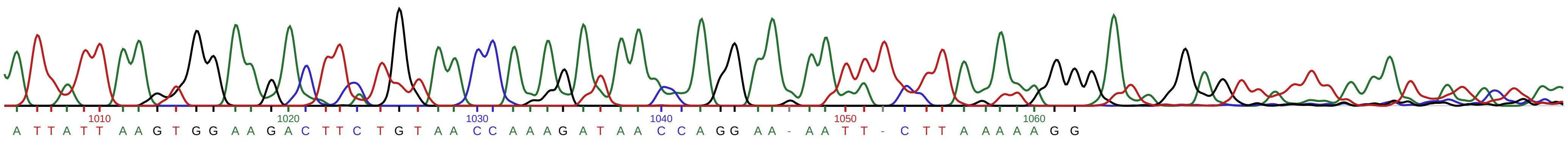

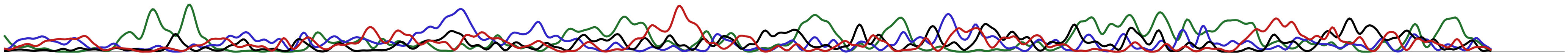

Supplement: Supporting information 2 — (ZIP) [file pone.0316479.s002.zip › 039KN2F_PREMIX_Plate_KELCH1_H06.pdf]
